# Rapid colorimetric detection of Zika virus from serum and urine specimens by reverse transcription loop-mediated isothermal amplification (RT-LAMP)

**DOI:** 10.1371/journal.pone.0185340

**Published:** 2017-09-25

**Authors:** Amanda E. Calvert, Brad J. Biggerstaff, Nathan A. Tanner, Molly Lauterbach, Robert S. Lanciotti

**Affiliations:** 1 Arboviral Diseases Branch, Division of Vector-Borne Diseases, U.S. Centers for Disease Control and Prevention, Fort Collins, CO, United States of America; 2 New England Biolabs, Ipswich MA, United States of America; University of California Davis, UNITED STATES

## Abstract

Zika virus (ZIKV) has emerged as a major global public health concern in the last two years due to its link as a causative agent of human birth defects. Its rapid expansion into the Western Hemisphere as well as the ability to be transmitted from mother to fetus, through sexual transmission and possibly through blood transfusions has increased the need for a rapid and expansive public health response to this unprecedented epidemic. A non-invasive and rapid ZIKV diagnostic screening assay that can be performed in a clinical setting throughout pregnancy is vital for prenatal care of women living in areas of the world where exposure to the virus is possible. To meet this need we have developed a sensitive and specific reverse transcriptase loop-mediated isothermal amplification (RT-LAMP) assay to detect ZIKV RNA in urine and serum with a simple visual detection. RT-LAMP results were shown to have a limit of detection 10-fold higher than qRT-PCR. As little as 1.2 RNA copies/μl was detected by RT-LAMP from a panel of 178 diagnostic specimens. The assay was shown to be highly specific for ZIKV RNA when tested with diagnostic specimens positive for dengue virus (DENV) and chikungunya virus (CHIKV). The assay described here illustrates the potential for a fast, reliable, sensitive and specific assay for the detection of ZIKV from urine or serum that can be performed in a clinical or field setting with minimal equipment and technological expertise.

## Introduction

Zika virus (ZIKV) is a mosquito-borne virus from the genus *Flavivirus* in the family *Flaviviridae*. Other notable viruses in this genus are dengue 1–4 viruses (DENV1-4), yellow fever virus (YFV), West Nile virus (WNV) and Japanese encephalitis virus (JEV). ZIKV was first isolated in 1947 from the blood of a febrile sentinel rhesus monkey during a yellow fever study in the Zika forest, Uganda[1]. Until recently, ZIKV was only known to cause a mild febrile illness with very few cases reported since its discovery. In April 2007, ZIKV caused an epidemic of disease in Yap State, Federated States of Micronesia[2], after which the virus spread across the South Pacific for the next 8 years before being identified as the causative agent of an outbreak of disease in March 2015 in Bahia, Brazil [3–5]. Since this time ZIKV has been confirmed to be transmitted sexually, from mother to fetus, and possibly through blood transfusions [6–11]. It has also been associated with microcephaly and other congenital brain abnormalities in some percentage of infants born to mothers infected during pregnancy [3, 5, 12, 13], as well as triggering cases of Guillain-Barre syndrome in infected patients [14, 15].

Laboratory diagnosis of ZIKV relies on the detection of viral RNA or anti-ZIKV specific IgM by ELISA or anti-ZIKV specific neutralizing antibody by plaque reduction neutralization test (PRNT). A definitive diagnosis based on serology or molecular assays is possible during primary ZIKV infections, but due to the cross-reactivity of flaviviruses during secondary infections a definitive diagnosis based on serology results alone is challenging to make[2, 5]. Some data suggests that ZIKV RNA may be detected over a longer period of time in urine than in serum [16–19]. The mean duration of viremia in serum by analysis of blood donations from blood centers in Puerto Rico in 2016 is estimated to be 10 days, while ZIKV RNA may be detected in serum from pregnant women up to 10 weeks after symptom onset [20, 21]. Since the primary way ZIKV infections can be definitively diagnosed is through the detection of ZIKV RNA during the acute phase of infection, a rapid diagnostic screening assay that can be performed throughout pregnancy in a clinical setting is vital for prenatal care of women living in areas of possible transmission of the virus.

Loop-mediated isothermal amplification (LAMP), developed by Notomi *et al*. (2000), is a technique for the rapid amplification of nucleic acid at a single constant temperature, typically 65°C. The isothermal nature of LAMP allows use of simple heating instruments, unlike PCR which requires specialized equipment for temperature cycling. LAMP uses 4–6 primers designed to amplify the gene target through creation of stem-loop structures that facilitate synthesis of new DNA by a strand-displacing DNA polymerase. These primers and loop structures create multiple initiating sites in the growing DNA products, enabling extremely rapid amplification. LAMP is also highly specific, since several primers and regions of homology are used to amplify a specific nucleic acid sequence[22]. Reverse-transcription LAMP (RT-LAMP) has been developed for the rapid detection of several mosquito-borne viruses in humans and mosquito pools including DENV, WNV, YFV, chikungunya virus (CHIKV), Rift Valley fever virus (RVFV), St. Louis encephalitis virus (SLEV) and Western equine encephalitis virus [23–30]. Here, we describe the development of an RT-LAMP assay for rapid screening for ZIKV RNA from urine and serum. Utilizing reagents from New England Biolabs, we developed an assay relying on a pH indicator in a low-buffer formulation for colorimetric detection of nucleic acid amplification[31]. As LAMP proceeds and creates nascent DNA, a proton is released from each dNTP incorporation, lowering the pH of the reaction mixture. With the pH indicator phenol red included in the LAMP mixture, the pH drop produced by amplification results in a change in the color of the reaction from pink to yellow. The ZIKV assay described here illustrates the potential use of this technology for the development of a fast, reliable, sensitive and specific assay for the detection of ZIKV from urine or serum that can be performed in a clinical or field setting with minimal equipment and technological expertise.

## Materials and methods

### Viruses

The following viruses utilized in this study were obtained from the Diagnostic and Reference Laboratory, Arboviral Diseases Branch, Division of Vector-Borne Diseases (Fort Collins, CO), or the Dengue Branch, DVBD (San Juan, Puerto Rico). ZIKV, strain PRVABC59, was isolated from a human infected in Puerto Rico in 2015. DENV1, strain R99142, was isolated from a traveler who visited Guatemala in 2013. DENV2, strain PR65-98, was isolated from a human in 1998 in Puerto Rico. DENV3, strain 100345, was isolated from a traveler who visited Nicaragua in 2014. DENV4, strain CAREC 08–10822, was isolated from a human specimen from St. Vincent, US Virgin Islands in 2008. CHIKV, strain 103268b, was isolated from a traveler who visited Bolivia in 2015. WNV, strain NY99-35262-11 was isolated from a flamingo at the Bronx zoo, New York, NY in 1999. SLEV, Strain MSI-7, was isolated from a house sparrow in Indianola, MS in 1975.

### Diagnostic specimens

The study obtained ethics approval for use of previously collected human diagnostic specimens from the U.S. Centers for Disease Control’s Human Subjects Internal Review Board (CDC IRB number: 6953). A total of 178 acute diagnostic specimens (urine: 84, serum: 94) randomly selected from patients with a ZIKV infection tested at the CDC by the Trioplex assay (https://www.fda.gov/downloads/MedicalDevices/Safety/EmergencySituations/UCM491592.pdf) for the detection of ZIKV, DENV1-4, and CHIKV were included in the study. A total of 68 diagnostic specimens (urine: 27, serum: 41) from patients testing negative for ZIKV infection were also included. Diagnostic specimens were de-identified for this study so that no link could be made between patient identification and assay result in this study.

### RT-LAMP primer design

ZIKV-specific RT-LAMP primers were designed using the nucleotide sequence of strain PRVABC59 (GenBank accession no. KU501215.1) and PrimerExplorer V5 software (http://primerexplorer.jp/e). Fifteen different primer sets were designed, and 3 primer sets with the most promising initial amplifications were evaluated in the RT-LAMP assay (Table 1). Primer set 1–1 amplifies the region in the ZIKV genome from nucleotides 1626 to 1849 included in the envelope (E) gene. Primer set 2–5 amplifies the region in the ZIKV genome between nucleotides 3682 to 3873 in the non-structural 2a (NS2a) gene. Primer set 5–5 amplifies the region in the ZIKV genome from nucleotides 7901 to 8143 included in NS5 gene.

**Table 1:** Primers used in RT-LAMP and qRT-PCR.

Primer	Sequence
**F3 1–1**	TGGTTCCACGACATTCCATT
**B3 1–1**	CATTTCAAGTGGCCAGAGGA
**FIP 1–1**	GGCATGTGCGTCCTTGAACTCTGACACCGGAACTCCACACT
**BIP 1–1**	AGAAGGAGCAGTTCACACGGCCCCTTTGCACCATCCATCTC
**LF 1–1**	ACCAGTGCTTCTTTGTTGTTCC
**LB 1–1**	CCTTGCTGGAGCTCTGGAG
**F3 2–5**	AATGAGTGACCTGGCTAAGC
**B3 2–5**	AAAAGACACGAGGCCAAGG
**FIP 2–5**	CAGCGCCAGATGAGCTACATCTTTTTGATGGGTGCCACCTTC
**BIP 2–5**	AGCGGCATTCAAAGTCAGACCATTTCACGGGGTGTCCAATT
**LF 2–5**	CCTCCAGTGTTCATTTCCGC
**LB 2–5**	GCGTTGCTGGTATCTTTCATCTT
**F3 5–5**	GATCTTGGATGTGGCAGAGG
**B3 5–5**	TGCTTCTTCCACTTCAGGAC
**FIP 5–5**	CGGGTTCTTCATGACCAGGGCGTTACTACGTCGCCACCATC
**BIP 5–5**	TCCGTCTTAAGAGTGGGGTGGATGTCACACAGCAACGTGTC
**LF 5–5**	CCTTTCACTTCTTGAACTTTGCG
**LB 5–5**	CGTCTTTCATATGGCGGCTG
**Q1-1F**	GGAACTCCACACTGGAACAA
**Q1-1R**	AACCACGACAGTTTGCCTTT
**Q1-1P**	[6FAM]AGAGTTCAAGGACGCACATGCCA[BHQ1a-Q]

### RNA extraction and *in vitro* transcribed RNA controls

Viral RNA from samples was extracted from virus supernatant with a QIAmp Viral RNA kit (Qiagen) following the manufacturer’s protocols. To prepare *in vitro* transcribed RNA from the gene region amplified by the 1–1 RT-LAMP primer set used as a copy number control, the ZIKV consensus primers (F31-1 and B31-1) with the T7 promoter sequence (TAATACGACTCACTATAGGGAGA) added to the 5' end of F31-1 primer were used to amplify a 224 base segment of cDNA (Table 1). The same size segment of RNA was transcribed from the cDNA using the mMessage mMachine kit (Life Technologies) according to the manufacturer’s protocol. RNA was quantified using the RNA Analysis screen tape on the Agilent 4200 TapeStation, and RNA copy numbers/μl were calculated based on spectrophotometry readings.

Viral RNA from 300 μl of specimens from spiked serum and urine panels was extracted using the QIAmp Viral RNA kit and eluted in 60 μl of AVE buffer. In order to process diagnostic specimens quickly and efficiently, viral RNA was extracted from 300 μl of sample using the MagMAX Pathogen RNA/DNA sample preparation system (ThermoFisher Scientific) according to the manufacturer’s protocol for low-cell-content samples and eluted in 90 μl of AVE buffer.

### Quantitative reverse-transcription polymerase chain reaction (qRT-PCR)

ZIKV-specific RNA extracted from samples were analyzed in triplicate by qRT-PCR using the RNA standard described above. A 5 μl aliquot of each purified RNA sample was added to master mix from Quantifast Pathogen RT-PCR kit (Qiagen) containing primers and probe designed using the region amplified by the 1–1 RT-LAMP primer set described above. To each reaction, 0.25 μl of 100 μM forward primer Q1-1F, 0.25 μl of 100 μM reverse primer Q1-1R, and 0.15 μl of 25 μM probe Q1-1P were added (Table 1). The reactions were analyzed on a BioRad CFX96 instrument under the following conditions: 50°C for 20 min, 95°C for 15 min, followed by 45 cycles of 95°C for 15 sec, and 60°C for 30 sec with continuous fluorescence data collection. Average C_t_ values were calculated based on triplicate wells. If no amplification occurred a value of 40 was assigned to the well.

### RT-LAMP assay

RT-LAMP reactions were carried out in triplicate in a 25 μl volume containing 1.6 μM each of inner primers FIP and BIP, 0.2 μM each of outer primers F3 and B3, 0.4 μM each of loop primers FL and BL, and either 12.5 μl of 2X Colorimetric LAMP Master Mix (Cat. No. M1800, New England Biolabs) and 10 μl of RNA template, or 5.0 μl of a custom-made 5X Colorimetric LAMP Master Mix and 17.5 μl of RNA template [31, 32]. Reactions were incubated at 65°C between 25 and 40 minutes in a heat block before results were recorded.

### Statistical analysis

Estimates of sensitivity, specificity, and (diagnostic) likelihood ratios positive and negative, and accuracy of the ZIKV RT-LAMP assay were calculated based on the results from 110 positive and 68 negative samples by RT-PCR[33]. Wilson’s score 95% binomial confidence intervals (CI) were computed for sensitivity and specificity, and score CIs were computed for the likelihood ratios and predictive values[34]. Estimates of positive and negative predictive values were computed using the estimated likelihood ratios over the full range of pre-test probabilities of ZIKV infection, and these values were displayed graphically. Differences in RT-LAMP positivity by specimen type were evaluated using Fisher’s exact test (mid-p).

## Results

### Optimization and amplification efficiency of ZIKV RT-LAMP assay

Viral RNA extracted from ZIKV-infected Vero cell supernatant was used as a standard to determine optimal reaction conditions for the RT-LAMP assay. RNA concentrations of this standard were quantitated using qRT-PCR and determined to be 4.8 x 10^6^ RNA copies/μl. Varying dilutions of viral RNA from 100 to 0.625 RNA copy/μl were made and tested in the RT-LAMP assay using primer sets 1–1, 2–5, 5–5 and combinations (1-1/2-5, 1-1/5-5, 2-5/5-5, and 1-1/2-5/5-5) at 65°C. Reaction results were recorded between 25 to 40 minutes in 5 minute intervals. Detection of nucleic acid amplification was determined visually with a color change from pink to yellow indicating amplification of nucleic acid. The reaction was considered complete once the non-template control (NTC) wells began to change color. The duration of this color change occurred between 30 and 40 minutes and was dependent on each individual primer set. Results were recorded at the 5-minute time interval in which all NTC wells were still negative for the primer set evaluated. Reactions were considered positive when 2 of the 3 wells displayed a color change. Two or three independent tests conducted in triplicate were used to calculate the expected limits of detection for each primer set using methods detailed in the online supplement (S1 File). Primer sets were tested with both the 2X LAMP master mix (MM) commercially available and a custom-made 5XMM which allowed for an additional 7.5 μl of RNA to be added to the reaction.

A decrease in the expected limit of detection was observed for all primer sets in the reaction using 5XMM and subsequent higher volumes of viral RNA with one exception (primer set 5–5). Primer sets 1–1 and 1-1/2-5/5-5 had the greatest decrease in the expected limit of detection from 19.3 and 8.5 RNA copies/μl, respectively, using the 2XMM and 10 μl of RNA in the reaction to 7.2 and 2.1 RNA copies/μl, respectively, in the reaction containing 5XMM concentration and 17.5 μl of RNA. Primer set 1-1/5-5 also had a notable decrease in the expected limit of detection from 10.0 RNA copies/μl with 2XMM to 5.4 RNA copies/μl with 5XMM. Relatively similar values were recorded for primer set 5–5 (6.2 RNA copies/μl and 6.6 RNA copies/μl with 2XMM and 5XMM, respectively) and primer set 2–5 (3.9 and 3.4 RNA copies/μl with 2XMM and 5XMM, respectively). Using the 5XMM, primer sets 1-1/2-5 and 1-1/2-5/5-5 were found to have the lowest expected limit of detection in the assay at 2.1 RNA copies/μl (Table 2). Three primer sets (2–5, 1-1/2-5 and 1-1/2-5/5-5) were subsequently tested for their ability to detect viral RNA in a panel of virus-spiked human serum specimens.

**Table 2:** Estimates (95% CI) of mean ZIKV RT-LAMP assay limit of detection for each primer set at different temperatures.

Primer Set	Master Mix	
	2X	5X
**1–1**	19.3^*a*^ (9.5, 38.8)^*b*^	7.2 (3.9, 11.8)
**2–5**	3.9 (2.9, 5.0)	3.4 (2.5, 4.2)
**5–5**	6.2 (3.7, 9.7)	6.6 (3.6, 10.8)
**1-1/2-5**	4.7 (3.5, 5.8)	2.1 (1.4, 2.7)
**1-1/5-5**	10.0 (6.6, 13.1)	5.4 (3.4, 9.5)
**2-5/5-5**	4.3 (3.0, 5.5)	2.8 (2.3, 3.2)
**1-1/2-5/5-5**	8.5 (4.6, 15.4)	2.1 (1.4, 2.9)

^*a*^ RNA copies/μl. The mean of the expected limit of detection was determined using ZIKV viral RNA with quantitated RNA copies measured by qRT-PCR using the gene region amplified by primer set 1–1. Reactions were incubated at 65°C for 20–40 minutes before results were recorded.

^*b*^ 95% confidence interval

### Sensitivity of ZIKV RT-LAMP assay

To evaluate the sensitivity of the ZIKV RT-LAMP assay compared to qRT-PCR viral RNA was extracted from 300 μl of sample from a virus-spiked serum panel and tested with primer sets 2–5, 1-1/2-5 and 1-1/2-5/5-5. The RNA in each sample was quantitated by qRT-PCR and ranged from 2.8 to 1476 RNA copies/μl. C_t_ values were also recorded since the CDC’s qRT-PCR diagnostic assay relies on C_t_ cutoffs to report positive and negative diagnostic results. A positive result in the qRT-PCR is any average C_t_ value 38.0 or less from triplicate wells while a negative test result is any average C_t_ value over 38.0 from triplicate wells. A C_t_ value of 40.0 was assigned if no amplification occurred in one of the triplicate wells. C_t_ values in the serum panel for samples containing ZIKV RNA ranged from 27.6 to 36.3 (Table 3). All primer sets reacted similarly when tested with the virus-spiked serum samples. All primer sets were able to detect viral RNA in sample containing more than 50 RNA copies/μl. Only 1-1/2-5/5-5 was not able to detect viral RNA in the sample (#9) containing 12.5 RNA copies/μl. None of the primer sets were able to detect viral RNA in sample (#1) containing 2.8 RNA copies/μl. This is not surprising since the limit of detection of the assay was determine to be in the range of 2.7 and 3.9 RNA copies/μl using diluted viral RNA. No false positive results were detected for any of the negative samples included in the panel with any primer set (Table 3).

**Table 3:** Evaluation of sensitivity of RT-LAMP with ZIKV serum panel.

Sample	RNA copies/ μl^*a*^	CT value^*b*^	RT-LAMP^*c*^		
			2–5	1-1/2-5	1-1/2-5/5-5
1	2.8	36.3	-	-	-
2	0	>40.0	-	-	-
3	164	30.6	+	+	+
4	0	>40.0	-	-	-
5	104	31.3	+	+	+
6	55	32.1	+	+	+
7	1476	27.6	+	+	+
8	0	>40.0	-	-	-
9	12.5	34.2	+	+	-
10	544	29.0	+	+	+
11	368	29.5	+	+	+
12	0	38.9	-	-	-

^*a*^ RNA copies/μl in each sample were evaluated in qRT-PCR using *in vitro* transcribed RNA encompassing the gene region amplified by primer set 1–1.

^*b*^ Average C_t_ values of the quantitated serum panel based on primers from gene region amplified by primer set 1–1 (5 μl RNA/reaction).

^*c*^ sensitivity of RT-LAMP was evaluated with each primer set using 5X MM. Reactions were incubated at 65°C for 20–40 minutes before results were recorded as positive (+) or negative (-).

The assay was also evaluated using a panel of urine samples spiked with varying concentrations of ZIKV using primer sets 2–5 and 1-1/2-5 that had the most sensitivity when tested with the spiked serum panel. ZIKV RNA concentrations in the panel ranged from 1.2 to 7047 RNA copies/μl and CT values ranged from 23.1 to 35.5 for samples containing ZIKV RNA (Table 4). Similar results to the serum panel were obtained for the urine panel. When the assay was performed using primer sets 2–5 and 1-1/2-5 all samples containing ZIKV RNA except the samples with the least amount of RNA (sample #4, 1.2 RNA copies/μl and sample #2, 12 RNA copies/μl) were detected (Table 4). No false positive results were detected for any of the negative samples included in the panel with either primer set (Table 4).

**Table 4:** Evaluation of sensitivity of RT-LAMP with ZIKV urine panel.

Sample	RNA copies/ μl^*a*^	CT value^*b*^	RT-LAMP^*c*^	
			2–5	1-1/2-5
1	316	27.5	+	+
2	12	33.7	-	-
3	44	30.3	+	+
4	1.2	35.5	-	-
5	0	41.4	-	-
6	0	39.9	-	-
7	7047	23.1	+	+
8	0	39.1	-	-

^*a*^ RNA copies/μl in each sample were evaluated in qRT-PCR using RNA transcript made within the gene region amplified by primer set 1–1.

^*b*^ Average C_t_ values of the quantitated urine panel based on primers from gene region amplified by primer set 1–1 (5 μl RNA/reaction).

^*c*^ Sensitivity of RT-LAMP was evaluated with each primer set using 5X MM. Reactions were incubated at 65°C for 20–40 minutes before results were recorded as positive (+) or negative (-).

A panel consisting of a variety of diagnostic specimens including whole blood, serum, and urine with and without nucleic acid preservative was also evaluated in the RT-LAMP assay with primer set 1-1/2-5 to determine the sensitivity and specificity of the assay compared to qRT-PCR results. The sensitivity of the qRT-PCR described here was validated in a separate experiment with quantitated viral RNA and displayed an equivalent sensitivity compared to the CDC’s single-plex real-time RT-PCR assay. RNA was extracted from 300 μl of sample and eluted in 100 μl of AVE buffer. Results from the CDC’s single-plex real-time RT-PCR assay for the detection of ZIKV using 20 μl of RNA and the ZIKV RT-LAMP assay using 17.5 μl of RNA were compared. RNA concentrations using the qRT-PCR along with the C_t_ values for this PCR were included in the analysis for each sample (Table 5). The ZIKV RT-LAMP assay was comparable to the CDC’s single-plex qRT-PCR when tested with the specimen panel with only two false negative samples recorded (#2, 3.3 RNA copies/μl and #8 9.6 RNA copies/μl). The same RNA used in the single-plex real-time RT-PCR was used for the LAMP assay and the PCR for quantitation, but had been freeze-thawed between uses possibly resulting in degradation of the RNA sample which may account for the drop in C_t_ values between the two PCR tests for all samples and the negative reactions in the ZIV RT-LAMP for samples #2 and #8. Overall, when compared to the single-plex real-time RT-PCR the RT-LAMP assay had a sensitivity of 80% (95% CI 49.0–94.3%) and specificity of 100% (95% CI 75.7–100%). While the sample size was low these results gave us sufficient confidence that the ZIKV RT-LAMP assay should perform relatively well with a large set of diagnostic specimens when compared to the qRT-PCR assay.

**Table 5:** Evaluation of performance of RT-LAMP with a proficiency panel of diagnostic specimens.

Sample	CT value CDC single-plex^*a*^	Ct value 1–1^*b*^	RNA copies/ μl^*c*^	RT-LAMP^*d*^
1	>40.0	>40.0	0	-
2	36.4	37.4	3.3	-
3	>40.0	>40.0	0	-
4	29.7	33.3	47.8	+
5	38.0	>40.0	0	-
6	30.5	32.7	71.2	+
7	>40.0	>40.0	0	-
8	32.5	35.4	9.6	-
9	31.3	36.7	11.1	+
10	32.3	34.5	18.5	+
11	>40.0	>40.0	0	-
12	32.4	36.5	5.2	+
13	38.1	>40.0	0	-
14	>40.0	>40.0	0	-
15	>40.0	>40.0	0	-
16	30.5	33.7	35.1	+
17	>40.0	>40.0	0	-
18	31.9	35.3	10.3	+
19	38.3	>40.0	0	-
20	28.9	32.0	124.6	+
21	>40.0	>40.0	0	-
22	>40.0	>40.0	0	-

^a^ Average C_t_ values based on the CDC RT-PCR single-plex assay using freshly extracted RNA run in duplicate wells for the detection of ZIKV.

^*b*^ Average C_t_ values of the qRT-PCR after freeze-thawing of RNA based on primers from gene region amplified by primer set 1–1 (5 μl RNA/reaction).

^*c*^ RNA copies/μl in each sample were evaluated in qRT-PCR after freeze-thawing of RNA using RNA transcript made within the gene region amplified by primer set 1–1.

^*d*^ sensitivity of RT-LAMP was evaluated with primer set 1-1/2-5 and 5X MM. Reactions were incubated at 65°C for 20–40 minutes before results were recorded as positive (+) or negative (-).

### Specificity of ZIKV RT-LAMP assay

**Fig 1:**
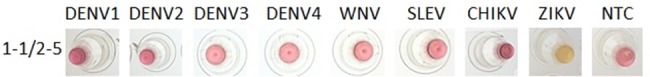
Visual detection of specificity of ZIKV RT-LAMP assay with the arbovirus urine panel. Photos represent 1 of 3 replicates. Reactions were incubated at 65°C for 40 minutes before results were recorded. DENV1, dengue virus 1, DENV2, dengue virus 2; DENV3, dengue virus 3; DENV4, dengue virus 4; WNV, West Nile virus; SLEV, St. Louis encephalitis virus; CHIK, chikungunya virus; ZIKV, zika virus; NTC, non-template control.

In order to estimate the specificity of the assay to detect only ZIKV RNA, a panel of urine samples spiked with varying concentrations of arboviruses including DENV1 (1.1 log_10_PFU/μl), DENV2 (4.9 log_10_PFU/μl), DENV3 (0.2 log_10_PFU/μl), DENV4 (0.3 log_10_PFU/μl), WNV (3.6 log_10_PFU/μl) SLEV (4.1 log_10_PFU/μl), and CHIKV (0.1 PFU/μl) were tested. RNA was extracted from 300 μl of spiked urine and tested in the assay using primer set 1-1/2-5 under the same conditions as previously described. The ZIKV RT-LAMP assay was shown to be highly specific for ZIKV since none of the spiked urine samples with other arboviruses were positive in the assay (Fig 1). Additionally, A panel of diagnostic specimens which tested positive in the CDC’s diagnostic Trio-plex RT-PCR assay for DENV (n = 10) with C_t_ values ranging from 15.77 to 31.92 or CHIKV (n = 10) with C_t_ values ranging from 18.47 to 24.23 were negative when tested in the ZIKV RT-LAMP assay.

### Evaluation of RT-LAMP assay for clinical diagnosis of ZIKV

**Fig 2:**
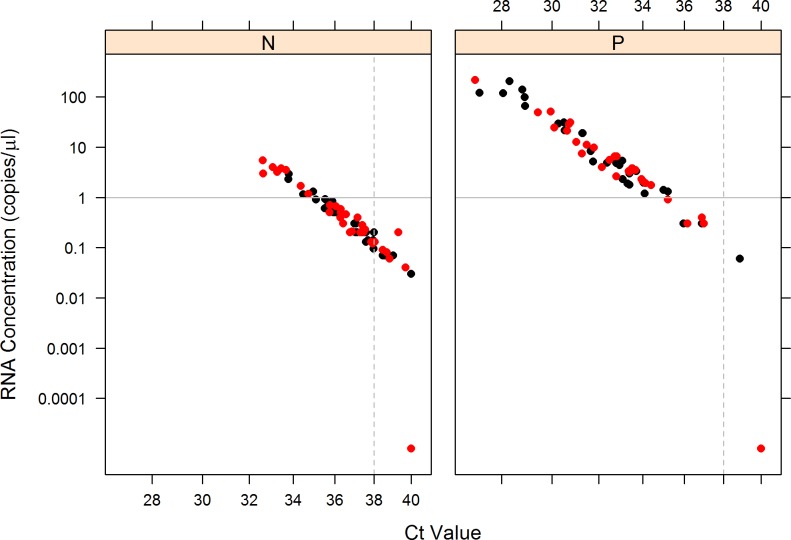
Evaluation of ZIKV RT-LAMP assay with diagnostic specimens. Negative (N) ZIKV RT-LAMP results and positive (P) ZIKV RT-LAMP results were separated and plotted based on Ct values (x-axis) and RNA concentrations (y-axis) from the corresponding RT-PCR. Urine samples are represented by black diamonds. Serum samples are represented by red diamonds. The solid horizontal line represents the 1 RNA copy/μl cutoff value. The dashed vertical line represents the Ct value 38.0. All Ct values ≥ 38.0 are considered negative in the RT-PCR.

**Fig 3:**
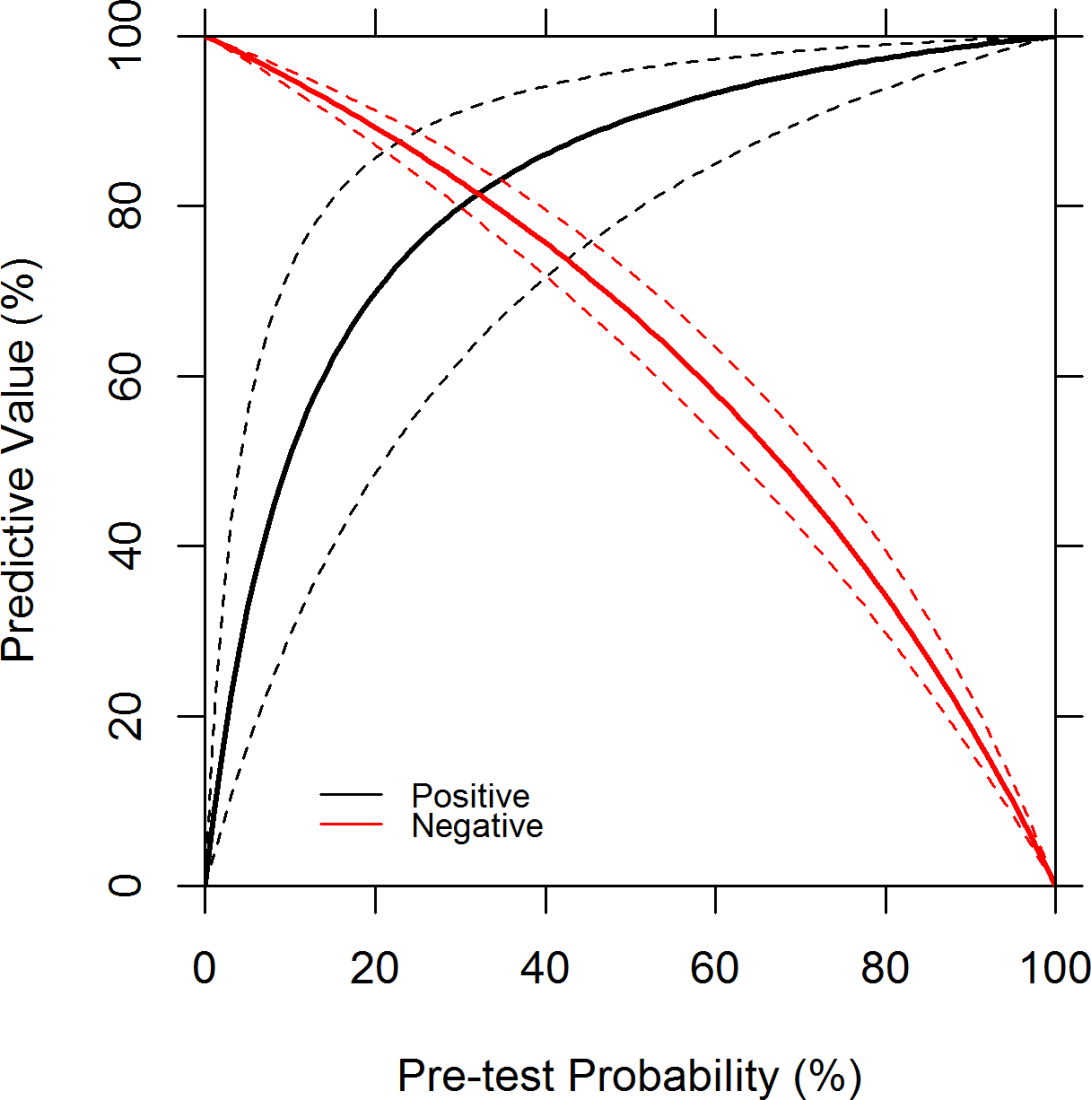
Predictive values for the ZIKV RT-LAMP assay based on pre-test probability percentages of ZIKV infection. Positive (solid black line) and negative (solid red line) predictive values (%), and 95% Cis (positive: dashed black line, negative: dashed red line), plotted against pre-test probability (%) for the RT-LAMP assay using all diagnostic samples combined.

A total of 178 diagnostic specimens (94 serum specimens and 84 urine specimens) were collected for testing in the ZIKV RT-LAMP assay. Of these, 110 were positive for ZIKV by qRT-PCR (53 ZIKV-positive serum samples, 57 ZIKV-positive urine samples), and 68 were negative by qRT-PCR (41 ZIKV negative serum samples, 27 ZIKV negative urine samples). The lowest RNA concentrations detected by qRT-PCR were 0.14 and 0.2 RNA copies/μl in serum and urine specimens, respectively. When diagnostic specimens had RNA concentrations <1.0 copy/μl (Ct values ranging 35.09 to 40.00) the ZIKV RT-LAMP assay was only able to detect 6 of 43 positive samples, a sensitivity of only 14.0% (95% CI 6.6–27.3%), while 64 of 68 RT-PCR negative samples were RT-LAMP negative, for a specificity of 94.1% (95% CI 85.8–97.7%). The likelihood ratio positive was 2.5 (95% CI 0.8–7.4), and the likelihood ratio negative was 0.9 (95% CI 0.8–1.0). When diagnostic specimens had RNA concentrations >1.0 copy/μl (Ct values ranging 35.20 to 24.23) the ZIKV RT-LAMP assay was able to detect 54 out of 67 positive samples, a sensitivity of 80.6% (95%CI 69.6–89.3%); there were no RT-PCR negative samples, so specificity and the likelihood ratios could not be estimated. These results are summarized in Fig 2 which separates negative and positive RT-LAMP results and plots them based on the C_t_ value from the real-time RT-PCR and RNA concentration. When all diagnostic samples were included in the statistical analysis, the assay had a sensitivity of 54.5% (95% CI 45.2–63.5%), specificity of 94.1% (95% CI 85.8–97.7%), likelihood ratio positive of 9.3 (95% CI 3.8–23.9) and likelihood ratio negative of 0.5 (95% CI 0.4–0.6). The overall accuracy of the ZIKV RT-LAMP assay was 69.7% (95% CI 62.6–75.9%) (Table 6). Fig 3 shows the predictive values (and 95% CIs) for the ZIKV RT-LAMP assay based on pre-test probability percentages of ZIKV infection. When the pre-test probability of ZIKV infection in the population was plotted against the positive and negative predictive values of the ZIKV RT-LAMP assay, the test is estimated to maximize both predictive values simultaneous when the pre-test probability of disease is approximately 30% (Fig 3). No statistical difference in RT-LAMP positivity by specimen type were detected in either PCR positive (Fisher’s exact mid-p = 0.9) or PCR negative (Fisher’s exact mid-p = 0.7) samples.

**Table 6:** The diagnostic performance of RT-LAMP based on RNA concentrations in the specimens.

RNA copies/μl	Sensitivity (%)	Specificity (%)	Likelihood Ratio Positive	Likelihood Ratio Negative	Accuracy (%)
<1.0	14.0 (6.6–27.3) *	94.1 (85.8–97.7)	2.5 (0.8–7.4)	0.9 (0.8–1.0)	63.1 (53.8–71.5)
>1.0	80.6 (69.6–89.3)	N/A**	N/A**	N/A**	80.6 (69.6–88.3)
total	55.4 (45.2–63.5)	94.1 (85.8–97.7)	9.3 (3.8–23.9)	0.5 (0.4–0.6)	69.7 (62.6–75.9)

*95% confidence interval

**No samples were RT-PCR negative

## Discussion

This report describes the development of RT-LAMP for the rapid detection of ZIKV in urine and serum and the usefulness of the colorimetric technology to be integrated into a point of care test. The ZIKV RT-LAMP assay demonstrated a level of detection similar to qRT-PCR when the Ct value was below 35.2 corresponding to an RNA concentration of ≥1.0 RNA copies/μl resulting in an accuracy rate of 80.6%. When samples included the analysis of those with an RNA concentration of <1.0 RNA copies/μl the accuracy of the diagnostic test dropped to 69.7%. The ZIKV RT-LAMP assay also demonstrated a high degree of specificity with only a 2.2% false positive rate calculated from negative diagnostic specimens tested in the assay; however the false negative rate was 27.5%.

While the false negative rate is fairly high in the ZIKV RT-LAMP assay, these are promising results for the development of a rapid point of care test for clinical screening of suspected ZIKV infections or routine testing for ZIKV infections in asymptomatic pregnant women. RT-LAMP offers an easy to use, convenient and cost-effective alternative to laboratory-based testing, particularly in field or clinical settings where the rapidity and convenience of screening diagnostic samples may outweigh the need for definitive diagnoses. Several groups have recently developed prototypes for ZIKV using RT-LAMP and other isothermal methods including recombinase polymerase amplification (RPA), nucleic acid sequence based amplification (NASBA) and RAMP (rapid amplification), a combination of LAMP and RPA techniques [35–43]. Tian et al (2016) developed RT-LAMP assay based on AC susceptometry in a portable reaction container for use in field settings. The limit of detection of this assay was determined to be 1 aM when using serum spiked with synthetically derived oligonucleotides, a measurement of RNA concentration we are unable to directly compare to our results [35]. Song et al (2016) also developed a portable RT-LAMP assay with colorimetric detection of ZIKV RNA sensitive enough to detect 5 plaque forming units of ZIKV in spiked saliva samples [39]. Detection of ZIKV by RT-LAMP using turbidity and color-change to detect nucleic acid amplification of ZIKV RNA was shown to have a limit of detection of 20 RNA copies per reaction or 4 RNA copies/μl [36], a similar limit of detection to results reported here. Others have developed RT-LAMP assays for simultaneous detection of ZIKV, CHIKV and DENVs using a self-contained heating device and smartphone for detection of nucleic acid amplification [38, 44]. These assays may offer fast and convenient methods for detection of ZIKV outside the laboratory setting, but their validation has focused on spiked samples which may or may not correlate with the limit of detection of viral RNA in real clinical specimens. In this study the median value of RNA concentration from specimens detected by qRT- PCR was 1.92 RNA copies/μl (a median Ct value of 34.09). These specimens and values for qRT-PCR are typical of diagnostic specimens tested for ZIKV at the CDC’s Diagnostic Labs, and we believe this study accurately demonstrates the level of sensitivity that can be obtained with RT-LAMP.

RT-LAMP assays have also been developed for a variety of arboviruses including dengue, chikungunya, yellow fever, Japanese encephalitis, St. Louis encephalitis, West Nile, and western equine encephalitis viruses [23, 30, 44–48]. The sensitivity of some of these RT-LAMP assays were reported to be much higher than conventional RT-PCR and similar to real time RT-PCR but without the need for expensive, sophisticated equipment [46, 48]. This discrepancy may be explained by a higher rate of false positive results known to occur in RT-LAMP. Our results and others have found RT-LAMP to have a limit of detection 10-fold higher than real time RT-PCR [23, 30, 36]. Our estimates of the expected limits of detection (95% CI) provide a quantitative assessment of assay sensitivity; however, we caution that for some of the primer sets the estimates are less well-estimated than others largely due to small sample sizes and the coarse interval censored nature of dilution assays which is reflected in the relatively wide CIs. Refinement of the estimates of expected limits of detection may be undertaken by increasing the numbers of replicates and/or increasing the number of possible dilutions.

While the ZIKV RT-LAMP assay developed here may be sensitive as or more sensitive than other RT-LAMP assays developed for ZIKV RNA detection there are several improvements that can be made to increase the sensitivity of the assay to detect those samples with RNA concentrations below 1.0. The sensitivity of LAMP has been shown to increase with an overall increase in the reaction volume[49]. Here we’ve shown that by changing the master mix of the reaction from 2X to 5X allowing more RNA input dramatically increased the sensitivity of the assay. In the case of primer set 1–1, that increase in sensitivity was measured to be more than 2 times higher in the reaction using the 5X MM over the 2X version. Increasing RNA input has also been shown to increase sensitivity in molecular diagnostic tests for the detection of ZIKV [50]. Concentrating virus for RNA extraction from a larger sample volume through virus particle capture can also increase the RNA concentration in the RT-LAMP assay thereby increasing assay sensitivity [51, 52]. Liu et al (2011) reported the development of a reaction cassette integrated with a membrane for isolation, concentration and purification of DNA and RNA. In this study nucleic acid captured directly by the membrane was used as template without an elution step eliminating inhibitors that may lower the sensitivity of the assay[51]. Others have used glass fiber membrane for DNA extraction of HIV-1 from blood samples[52]. The direct detection of viral RNA without RNA extraction has also been reported for ZIKV [37]. Others have reported that nucleic acid extraction may not be necessary with some pathogens if the temperature is high enough in the reaction to allow the virion to become permeable by primers and enzymes providing access to viral RNA. The enzymes utilized in LAMP are more resistant to inhibitory components in clinical specimens [44, 53–56].

Real time RT-PCR remains the most sensitive method for detection of ZIKV RNA from human specimens in a diagnostic laboratory setting; however, the direct comparisons of the ZIKV RT-LAMP assay and the qRT-PCR with equivalent sensitivities to the CDC’s single-plex assay indicate that this assay has the potential to be used for diagnostic screening of ZIKV in a clinical setting. We propose that using a rapid point-of-care test such as the one reported here can greatly enhance prenatal care of women living in areas where ZIKV may circulate. By utilizing this diagnostic screening tool for the detection of ZIKV infections in asymptomatic pregnant women throughout pregnancy more information will be available to health care providers and patients during critical times of care.

## Supporting information

S1 File Estimated limit of detection analysis. A detailed description of the calculation for the estimated limit of detection is explained.
(DOCX)
