# Supplementary material for: Rapid colorimetric detection of Zika virus from serum and urine specimens by reverse transcription loop-mediated isothermal amplification (RT-LAMP)
Source: PLoS One. 2017 Sep 25;12(9):e0185340. doi: 10.1371/journal.pone.0185340 (PMC5612724; doi:10.1371/journal.pone.0185340)
Supplement: S1 File — A detailed description of the calculation for the estimated limit of detection is explained. (DOCX) [file pone.0185340.s001.docx]

S1 File. Estimated Limit of Detection Analysis.

At each master mix/primer set combination, the recorded RNA concentrations in this experiment represent a limit of detection for the individual well measurements, while the algorithm determining positivity of a sample—2 of 3 replicate wells positive—gives a limit of detection (LOD) for the assay as implemented. We estimate the expected LOD(s) of the assay from the reported experiment as we now describe. The data suggest that the LOD may vary by master mix and primer set, although because the available information limits our ability to model these experimental combinations simultaneously, we estimate the LODs separately for these. We describe the estimation approach generally, and then apply the same methods to each experimental condition.

Since the RNA concentration/dilution sequences differed for two different batches of sample, observations were recorded as one of batch 1 concentrations [1.06, 2.39, 3.74, 4.46, 13.18, 27.10, 345.05 RNA copies/µl] and batch 2 concentrations [0.47, 0.93, 1.67, 3.40, 6.00, 12.00, 116.00 copies/µl] for individual replicate measurements of RNA concentration. This mixing of concentrations and unequal dilution stepping made standard methods for dilution assays not readily applicable. Depending on the master mix/primer set combination, either 6 or 9 replicates were performed. These replicates were grouped in triplicates and the assay algorithm of identifying the second smallest value of the three as the final response for the triplicate set was used (if the smallest two were equal, this value was used), and this yielded either 2 or 3 observations of assay LOD per master mix/primer set combination. The distribution of this final response from the assay algorithm is of interest when making inference regarding the LOD for the assay, since this is the quantity that is used to characterize samples.

The distribution of the assay LOD may be derived from the distribution of the individual replicates by noting that the value of the second-largest observation is the second order statistic of three observations for independent replicates grouped into threes. A model for the distribution of the individual LOD replicates thus readily yields the required distribution for the assay LOD using standard results from the distribution theory of order statistics. Noting that individual replicate responses are reasonably modeled as continuous, positive, skewed right, and interval censored, we modeled these values using standard survival analysis methods assuming either a Weibull (when at least 4 unique values were available) or an exponential (fewer than 4 unique values available); recall the exponential distribution is a special case of the Weibull. Resulting shape and scale estimates therefore provide a model fit for the individual LOD replicates. Denote by $F(x; r, \lambda)$ the cumulative distribution function (CDF) of the Weibull distribution with shape parameter $r$ and scale parameter $\lambda$; $r$ is fixed at 1 for the exponential distribution. The resulting CDF of the distribution of the assay LOD is, therefore, $F_{2;3}(x;r,\lambda)= F\left( x; r, \lambda\right)^{2}\left[ 3-2F(x; r, \lambda) \right]$, where the subscript 2;3 indicates the second order statistic of a sample of size three.

Implementing the survival analysis approach described provides maximum likelihood estimates $\hat{r}$ and $\hat{\lambda}$ in the case of the Weibull, and $\hat{\lambda}$ for the exponential, and these may be used directly in the expression for $F_{2;3}(x;r, \lambda)$, giving an estimate of the distribution for the assay LOD. The expected assay LOD is thus the mean of this distribution, which must be computed numerically using the probability density function derived from $F_{2;3}(x;r, \lambda)$ and numerical integration. Confidence intervals for the mean assay LOD may theoretically be computed using asymptotic results, but with few observations here, instead we use bootstrap resampling (1). Briefly, the 6 or 9 individual replicates were resampled with replacement 10,000 times, and for each of these resample datasets, maximum likelihood estimates $\hat{r}$ and $\hat{\lambda}$ were computed and the mean of $F_{2;3}(x;\hat{r},\hat{\lambda})$ calculated. The average of these 10,000 bootstrap resample values of the mean assay LOD are our final estimates for the assay LOD, while the empirical 2.5th and 97.5th quantile points of the 10,000 bootstrap resample values provide a 95% confidence interval (CI) for the mean assay LOD. Estimates of the mean assay LOD (95% CI) are reported in Table 2.

1. Davison AC, Hinkley DV. Bootstrap methods and their application. Cambridge ; New York, NY, USA: Cambridge University Press; 1997.
